# P-1296. Impact of Metallo-Beta Lactamases on Outcomes in Patients with Carbapenem-Resistant Gram-Negative Infections

**DOI:** 10.1093/ofid/ofaf695.1484

**Published:** 2026-01-11

**Authors:** Angelique E Boutzoukas, Wanying Shao, Jianping Jiang, Souha S Kanj, Minggui Wang, Soraya Salcedo Mendoza, Kalisvar Marimuthu, Zhengyin Liu, Lauren Komarow, Vance G Fowler, Barry N Kreiswirth, Cesar A Arias, Yohei Doi, David L Paterson, Michael Satlin, Robert A Bonomo, David van Duin

**Affiliations:** Duke University/Duke Clinical Research Institute, Raleigh, NC; George Washington University, Rockville, Maryland; Center for Discovery and Innovation, Hackensack Meridian Health, Nutley, NJ; American University of Beirut Medical Center, Beirut, Lebanon; Institute of Antibiotics, Huashan Hospital, Fudan University, Shanghai, Shanghai, China (People's Republic); Facultad de Ciencias de la Salud, Universidad Simón Bolívar, Barranquilla, Colombia, Barranquilla, Atlantico, Colombia; National Centre for Infectious Diseases, Singapore, Tan Tock Seng, Not Applicable, Singapore; Infectious Disease Section, Department of Internal Medicine, Peking Union Medical College Hospital, Beijing, Beijing, China; George Washington University, Rockville, Maryland; Duke University Medical Center, Durham, NC; Center for Discovery and Innovation, Hakensack Meridian Health, Nutley, New Jersey; Houston Methodist and Weill Cornell Medical College, Houston, TX; University of Pittsburgh, Toyoake, Aichi, Japan; 3The University of Queensland, Queensland, Queensland, Australia; Weil Cornell Medicine, New York, New York; Case Western Reserve University/ Louis Stokes Cleveland VA Medical Center, Cleveland, OH; University of North Carolina at Chapel Hill, Chapel Hill, NC

## Abstract

**Background:**

Treatment options for Carbapenem-resistant (CR) Gram-negative infections due to metallo-beta-lactamase (MBL) enzymes are limited. The clinical impact of MBLs vs. other mechanisms of carbapenem resistance in Enterobacterales and non-fermenting bacteria remains unclear.

**Table 1.**
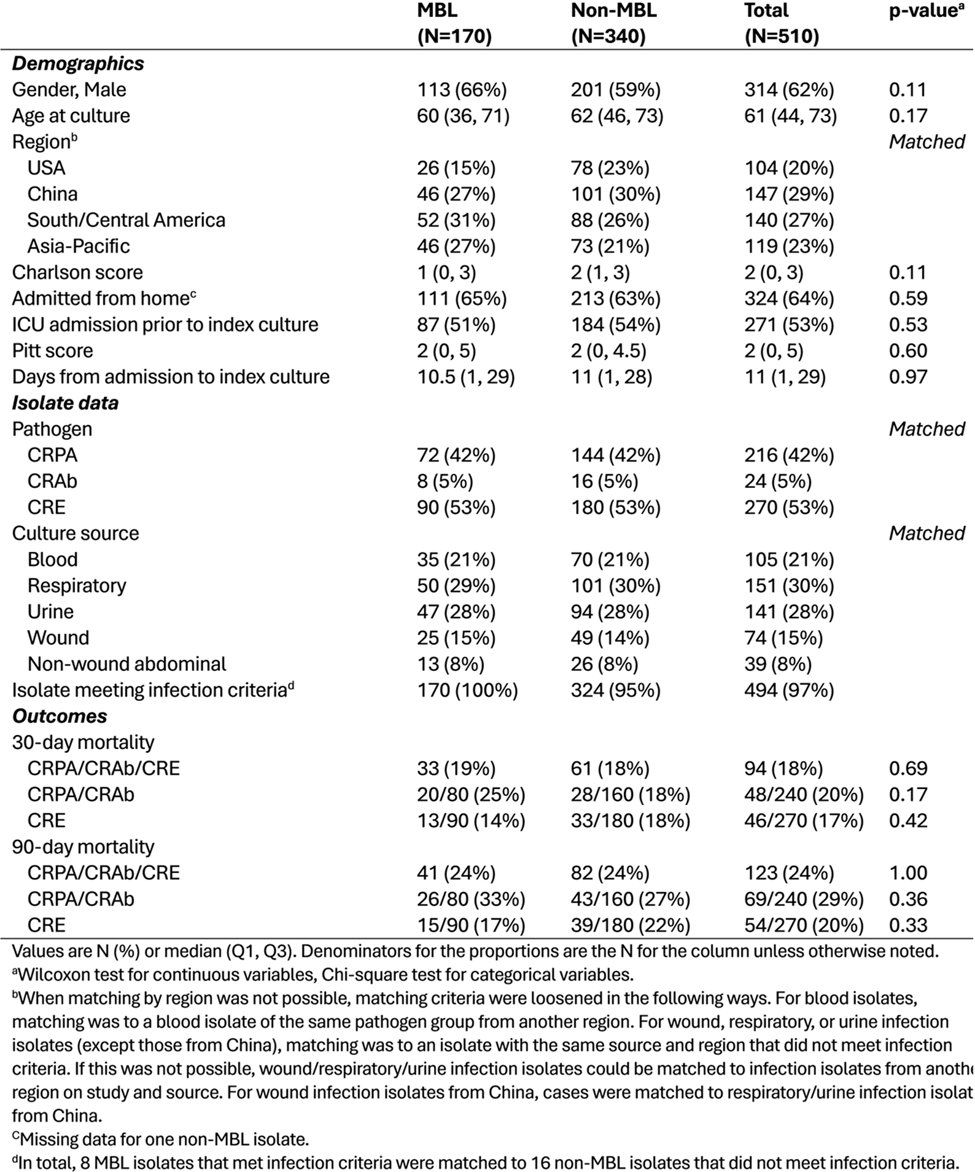
Demographics, isolate characteristics, and outcomes of patients with carbapenem-resistant Gram-negative infections, stratified by MBL status

**Figure 1.**
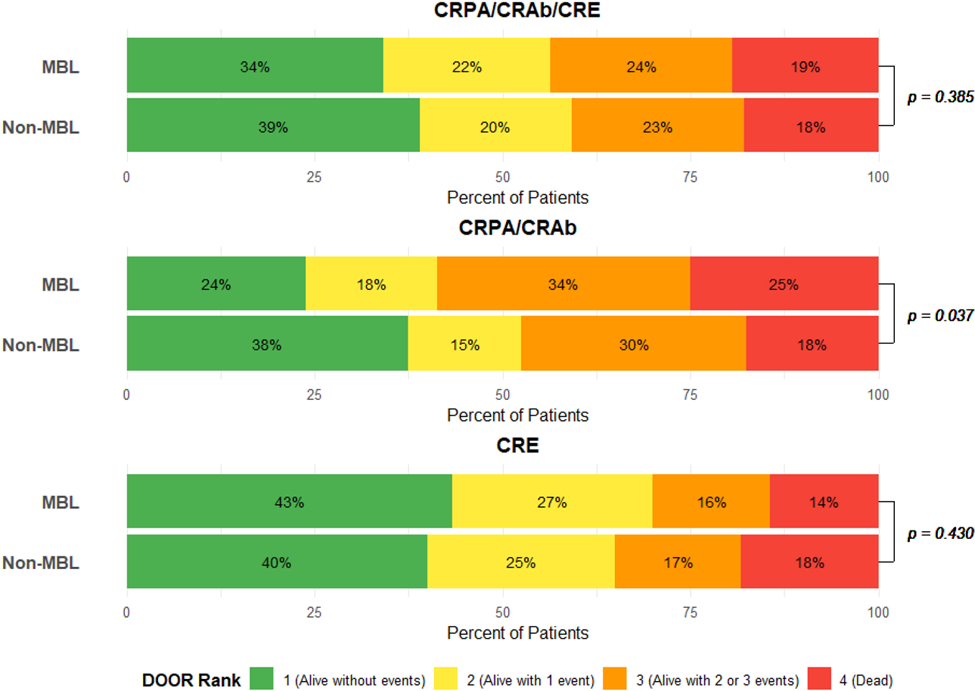
30-day desirability of outcome ranking (DOOR) outcomes of patients with carbapenem-resistant Gram-negative infections, stratified by MBL status CRPA – carbapenem-resistant Pseudomonas aeruginosa, CRAb – carbapenem-resistant Acinetobacter baumannii, CRE – carbapenem-resistant Enterobacterales, DOOR – desirability of outcome ranking. MBL – metallo-beta-lactamase. DOOR events assessed at 30 days include: unsuccessful discharge, lack of clinical response, and C. difficile infection and/or renal failure. Not all rows total to 100 due to rounding. P-value calculated using Wilcoxon test.

**Methods:**

We conducted a matched cohort study of patients enrolled in one of three MDRO Network studies, POP (CR *Pseudomonas aeruginosa* [CRPA]), SNAP (CR *Acinetobacter baumannii* [CRAb]), or CRACKLE-2 (CR-Enterobacterales [CRE]) with isolates that met infection criteria. Patients with MBL-producing isolates (*bla*_VIM_, *bla*_IMP_, or *bla*_NDM_ present) were matched 1:2 to patients with non-MBL CR isolates (a different carbapenemase or CR without a carbapenemase) based on study, region, and anatomical source. We compared baseline characteristics, 30- and 90-day mortality, and 30-day desirability of outcome ranking (DOOR) scores.

**Figure:**
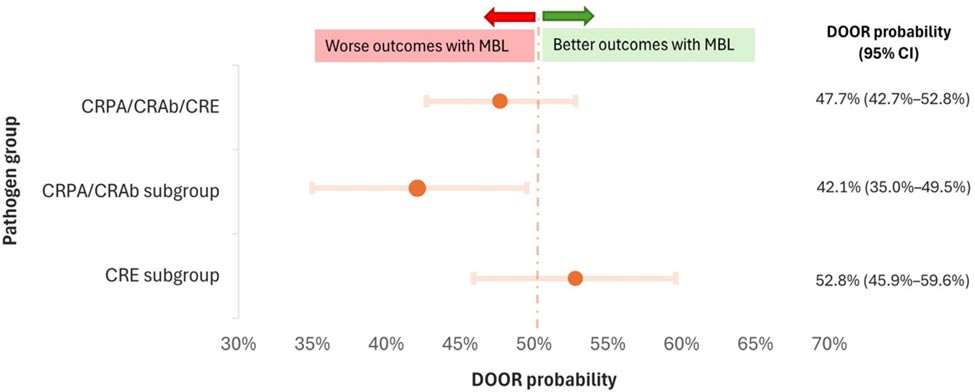
30-day Desirability of Outcome Ranking (DOOR) Probability by MBL Status Legend: CI – confidence interval, CRPA – carbapenem-resistant Pseudomonas aeruginosa, CRAb – carbapenem-resistant Acinetobacter baumannii, CRE – carbapenem-resistant Enterobacterales, DOOR – desirability of outcome ranking. MBL – metallo-beta-lactamase. The DOOR probability was calculated as the probability of a more desirable result in the presence of MBL as compared to non-MBL isolate. Confidence intervals were calculated using the method in Halperin et al (Biometric 1989; 45:500-521), and CI’s that do not include 50% are considered statistically significant. Estimates less than 50% signify a less favorable outcome for the MBL group, while estimates greater than 50% signify more favorable outcomes for the MBL group.

**Results:**

In total, 170 MBL isolates were matched to 340 non-MBL isolates from 10 countries (Table 1). The cohort included 42% CRPA (216/510), 5% CRAb (24/510), and 53% CRE (270/510). Demographics were balanced between groups; median age at culture was 61 (Q1, 44, Q3 73) years. Common infection sources were respiratory (151/510, 30%), urine (141/510, 28%), and blood (105/510, 21%). Of the MBL isolates, 92/170 harbored *bla*_NDM_ (54%), 62/170 harbored *bla*_VIM_ (36%), and 20/170 harbored *bla*_IMP_ (12%); four isolates co-harbored two distinct MBL enzymes. All-cause 30-day mortality was 19% (33/170) for MBL vs 18% (61/340) for non-MBL (p=0.69); MBL presence was not associated with 30- or 90-day mortality. DOOR outcomes at 30-days (Figure 1) did not differ by MBL status in the full cohort or the CRE subgroup, but did differ in the CRPA/CRAb subgroup (p=0.037). Among CRPA/CRAb infections, MBL presence was associated with less desirable outcomes (DOOR probability 42.1%; 95% Halperin confidence interval: 35.0%-49.5%, Figure 2).

**Conclusion:**

MBL presence was not associated with increased 30- or 90-day mortality compared to matched non-MBL isolates. However, in non-fermenter infections (CRPA/CRAb), MBL presence was linked to less desirable outcomes, an association not seen in CRE. These findings may inform prioritization of anti-MBL agents in future drug development.

**Disclosures:**

Angelique E. Boutzoukas, MD, MPH, Elion Therapeutics: Advisor/Consultant|Innoviva Speciality Therapeutics: DSMB Participant Souha S. Kanj, MD, Menarini: Honoraria|pfizer: Honoraria Vance G. Fowler, MD, MHS, Affinergy, Janssen, Contrafect: Advisor/Consultant|AstraZeneca; EDE; Basilea: Grant/Research Support|Debiopharm, GSK; Affinium, Basilea,: Advisor/Consultant|Destiny, Amphliphi, Armata, Akagera: Advisor/Consultant|Merck; Contrafect; Karius; Janssen: Grant/Research Support|UpToDate: Royalties|Valanbio: Stock options Yohei Doi, MD, PHD, GSK: Advisor/Consultant|Meiji Seika Pharma: Advisor/Consultant|Shionogi: Advisor/Consultant|Shionogi: Honoraria Michael Satlin, MD, MS, AbbVie: DSMB Participant|bioMerieux: Grant/Research Support|Merck: Grant/Research Support|SNIPRBiome: Grant/Research Support Robert A. Bonomo, MD, Merck: Grant/Research Support|Shinogi: Grant/Research Support|VenatoRx: Grant/Research Support David van Duin, MD, PhD, British Society for Antimicrobial Chemotherapy: Editor stipend|Merck: Advisor/Consultant|Merck: Grant/Research Support|Pfizer: Advisor/Consultant|Roche: Advisor/Consultant|Shionogi: Advisor/Consultant

